# Use of machine learning in predicting continuity of HIV treatment in selected Nigerian States

**DOI:** 10.1371/journal.pgph.0004497

**Published:** 2025-04-24

**Authors:** Mukhtar Ijaiya, Erica Troncoso, Marang Mutloatse, Duruanyanwu Ifeanyi, Benjamin Obasa, Franklin Emerenini, Lucien De Voux, Thobeka Mnguni, Shantelle Parrott, Ejike Okwor, Babafemi Dare, Oluwayemisi Ogundare, Emmanuel Atuma, Molly Strachan, Ruby Fayorsey, Kelly Curran

**Affiliations:** 1 Jhpiego - an Affiliate of Johns Hopkins University, Wuye District, Abuja, Federal Capital Territory, Nigeria; 2 Jhpiego - an Affiliate of Johns Hopkins University, Baltimore, Maryland, United States of America; 3 Palindrome Data, Cape Town, South Africa; 4 ICAP at Columbia University (Nigeria Country Office), Jabi, Abuja, Federal Capital Territory, Nigeria; 5 ICAP at Columbia University, New York City, New York, United States of America; Islamic Azad University South Tehran Branch, IRAN, ISLAMIC REPUBLIC OF

## Abstract

Nigeria, with the second-largest HIV epidemic globally, faces challenges in achieving its HIV epidemic control goals by 2030, with interruptions in treatment (IIT) a significant challenge. Machine learning (ML) models can help HIV programs implement targeted interventions to improve the quality of care, develop effective early interventions, and provide insights into optimal resource allocation and program sustainability. This paper aims to identify predictors and measure the performance of models used to predict the risk of IIT among People Living with HIV (PLHIV) on antiretroviral therapy (ART). We trained multiple supervised ML algorithms on de-identified client-level electronic medical records data from a cohort of PLHIV across four Nigerian states. Merged demographic, clinic, pharmacy, and laboratory data were included as potential predictor variables in multiple models. The study analyzed data from 41,394 PLHIV, with 266,520 observations receiving treatment across four Nigerian states. The overall IIT rate was 33.7%, ranging from 17.7% in Cross River State to 42.4% in Niger State. The AdaBoost model demonstrated the best performance, with a sensitivity of 69.2%, specificity of 82.3%, F1 score of 0.678, and PR-AUC and ROC-AUC values of 0.563 and 0.843, respectively. Key predictors included PLHIV prior behavior, visit history, and geographic factors, while demographic features played a lesser role. This study highlights the utility of ML, particularly the AdaBoost model, in stratifying PLHIV by the risk of IIT. By leveraging ML, HIV programs can implement data-driven, targeted interventions to improve care continuity. However, further research is needed to address data biases and contextual challenges in resource-constrained settings.

## Introduction

Approximately 1.9 million people live with HIV in Nigeria, making it the country with the second-largest HIV epidemic in the world [1]. It is estimated that there are 46,000 AIDS-related deaths each year in the country, with individuals aged 15 years and older accounting for 58% of these deaths [2]. In 2016, Nigeria adopted the World Health Organization’s (WHO) Test and Treat Strategy to ensure all PLHIV are placed on antiretroviral therapy (ART) irrespective of CD4 and clinical staging [3]. The lifelong use of ART is considered the standard of care for People Living with HIV (PLHIV) [4]. Despite the successful implementation of the Test and Treat Strategy in Nigeria, interruptions in treatment (IIT), defined as failure to make clinical contact for at least 28 days after the last expected visit, persist as a challenge to achieving the country’s HIV epidemic control goals by 2030 [5].

Reducing IIT is a priority for HIV intervention programs, as retention is critical for improving the health of R0Cs, preventing new infections, and reducing mortality and incidence of drug resistance [6,7]. In low- and middle-income countries, the rate of IIT is higher than the global average [8]. Significant factors associated with IIT include availability, accessibility, and cost of accessing care [9,10]. Furthermore, long waiting hours and poor care experience discourage PLHIV from attending their subsequent appointments, leading to IIT [11]. Moreover, HIV infection carries profound psychological and social implications, with many PLHIV prone to experiencing depression and stigma, undermining their retention in care [12,13]. Conversely, some PLHIV, often called having an IIT, have instead sought care in a different facility without filing transfer documentation [14].

Several innovations, including case management systems, tracking and call back, and clinic appointment diaries, minimize IIT, improve adherence and achieve viral suppression [15]. However, these strategies can be challenging to implement in resource-limited settings, requiring significant human and capital resources and correct and consistent documentation [16].

Nigeria’s HIV program now has adequate data with extensive client-level information from Electronic Medical Records (EMR), which offers the opportunity to use data and predictive analytics to identify individuals at high risk of experiencing IIT. HIV programs have recognized the need for more effective and targeted approaches to identify these high-risk individuals, which includes using data-driven and predictive methods [17]. Machine learning (ML), which uses statistical and computational algorithms, is an effective technique for unearthing complex relationships from vast amounts of data, allowing a deeper level of analysis than traditional statistics commonly used [18]. ML refers to the ability of systems to learn from training data to automate the development of analytical models [19]. These techniques can capture complex non-linear interactions between variables, supporting the complex factors that may result in an IIT [20,21]. These ML models can also help HIV programs implement targeted interventions to improve the quality of care, develop effective early interventions, and provide insights into optimal resource allocation and program sustainability [22].

However, there are currently few published manuscripts in this evolving field of ML in predicting IIT in resource-constrained settings such as Nigeria. This study hypothesizes that available client variables and features within the EMR can predict IIT among PLHIV in Nigeria. Furthermore, we postulate that ML models can outperform traditional statistical methods in detecting the intricate interplay between these variables. Consequently, we aim to identify and estimate the significance of these predictors for the PLHIV most at risk of IIT, and to assess the predictive accuracy of ML models.

## Methods

### Ethics statement

Based on the determination of Exempt Category (4) (ii) under 45 CFR 46 and the Revised Common Rule, this analysis was deemed exempt by the Johns Hopkins School of Public Health Institutional Review Board FWA #00000287, “Secondary Data Analysis of HIV Prevention, Care and Treatment Service Delivery Programs in Africa.” The study involved a retrospective secondary analysis of de-identified, routinely collected client-level data.

### Program description

The Reaching Impact, Saturation, and Epidemic Control (RISE) Program is a five-year global project funded by the U.S. President’s Emergency Plan for AIDS Relief (PEPFAR) through the U.S. Agency for International Development (USAID). In Nigeria, RISE works to address the challenges of HIV/AIDS and to reduce the burden of unmet needs. RISE began implementation in October 2019 and, at the time of the data extraction and analysis in June 2021, supported 101 health facilities across four states in Nigeria (Adamawa, Akwa Ibom, Cross River, and Niger). Both Akwa Ibom and Cross River states are in the South-South geopolitical zone of Nigeria. Adamawa is located in the Northeast geopolitical zone, and Niger State is in the North Central geopolitical zone. Akwa Ibom state has the highest HIV prevalence in Nigeria at 5.5%, with Adamawa, Cross River, and Niger at 1.2%, 2.0%, and 0.7%, respectively [1].

### Study population and data sources

**Fig 1:**
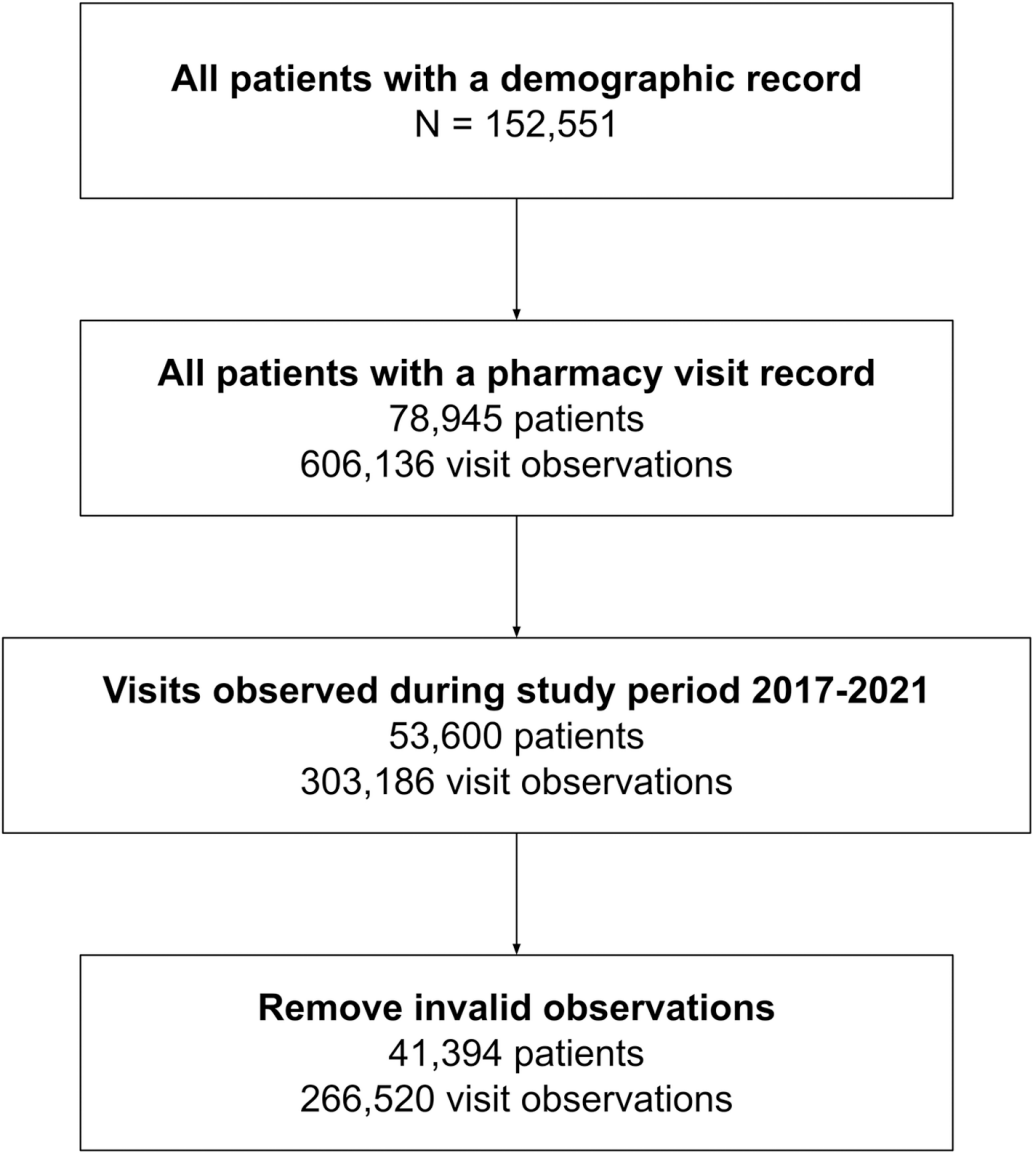
Flow chart of data source inclusion.

We analyzed PLHIV data across all 101 RISE-supported health facilities in Nigeria. Data tables were exported from the Lafiya Management Information System (LAMIS), an EMR database (Table 1). The earliest dated records were from 2002, with varying degrees of quality, including completeness, accuracy, and consistency, with its quality considerably improving from the mid-2010s onward. Therefore, we included PLHIV client records of only those who had started ART and had at least two pharmacy visits between Jan 1, 2017, and May 31, 2021. Fig 1 below illustrates the process of defining the PLHIV population for modeling.

**Table 1:** List of data tables.

Data Source	Description
Patient	Contains basic personal information about patients, such as date of birth, gender, and other baseline clinical information.
Laboratory	Contains longitudinal patient-level HIV viral load and CD4 results.
Pharmacy	Contain longitudinal patient-level pharmacy visits (drug pickups), date, and regimen information.
Clinic	Contains longitudinal patient-level clinic visits and appointment date information.

### Data preparation

The patient table was initially merged with the pharmacy table to create the master dataset. By joining the other data tables on exact dates or previous visits, we minimised the possibility of data leakage. A standardization procedure was carried out to fix varying formats and spelling differences. We excluded variables with a high proportion (>=50%) of missing values to enhance the dataset’s completeness. We did not conduct multiple imputations because the percentage of missing values for the other variables was below the threshold value of 5% and considered inconsequential to the quality of our analysis [23]. Furthermore, categorical and qualitative data were converted into a numerical format by creating columns for each category or data point.

### Feature engineering

The primary outcome of interest was an IIT, a missed clinic drug refill appointment of more than 28 days consecutively per PEPFAR guidelines [24].

All available data points from the demographics, clinic, pharmacy and laboratory datasets were considered in this analysis. Several features were created based on need, previous study literature, and the RISE team’s programmatic knowledge. We employed different approaches in building these features, including computation from baseline data and dates. This approach allowed for the derivation of a quantitative measure, such as PLHIV age, which could be used in subsequent analyses. In addition, a clinical feature such as *“weight loss”* was defined as a binary variable, denoting the presence or absence of a weight loss greater than 10% from the PLHIV previous clinic visit. Time-based features were extracted by leveraging the PLHIV most recent visit, drug pickup date, and scheduled appointment date. A complete list of engineered features is outlined in the supplementary file (S1 Table).

### Model development

After the tables were merged and the master dataset created, we adopted measures to address sampling bias and overfitting by randomly assigning PLHIV, rather than records, into the train (65.0%), test (19.0%), and validation sets (16.0%). This approach ensures the independence and generalizability of the model for future use [25]. To further minimize the risk of bias in the model’s performance, the primary outcome and sex distribution were balanced across the three sets. After that, we trained the models using three supervised ML algorithms: logistic regression, boosting (AdaBoost), and bagging (Random Forest). Logistic regression, a type of generalized linear model, is a common statistical method for analyzing binary outcomes [26]. AdaBoost and Random Forest are ensemble classification models that aggregate predictions from multiple base models to capture complex non-linear relationships [27]. The test set was used to evaluate the performance of the trained models. In tuning/optimizing the models, sensitivity, positive predictive value (PPV), and precision-recall under the curve (PR-AUC) were prioritized because the objective was to maximize the identification of PLHIV with IIT and minimize the cost of misclassifying an IIT.

**Fig 2:**
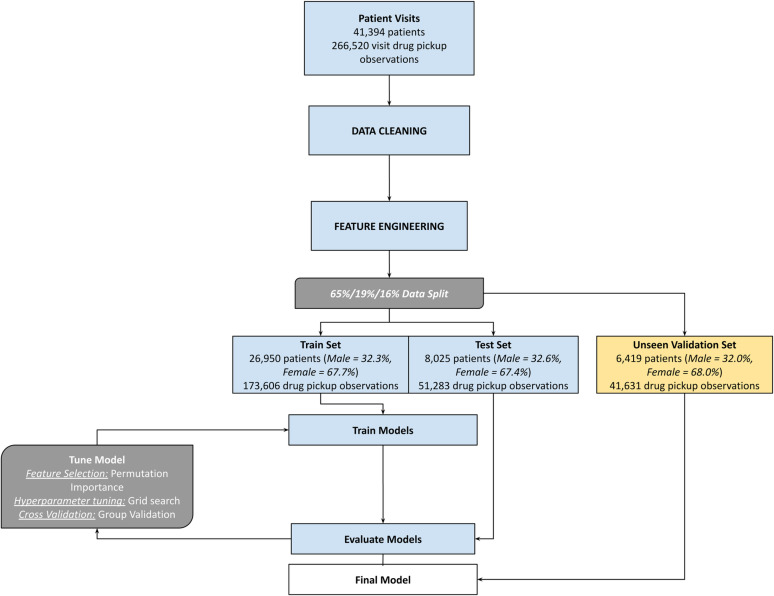
Predictive model-building process.

The most predictive features were selected for the model by removing highly colinear features and subsequently applying permutation feature importance (PFI) to the test set to determine which features had the most impact on the model’s predictions. PFI measures the increase in the model’s prediction error after permuting the feature’s values. We also included features important to the RISE program, such as demographics and Enhanced Adherence Counselling (EAC) enrolment. Hyperparameter tuning was performed on the AdaBoost model and its base estimator, a decision tree, using a grid search and cross-validation method to identify the optimal hyperparameters. Finally, the optimally tuned model was evaluated on the validation set to ensure it performed well on unseen data. All analyses were conducted using Python (version 3.8) [28]. Data preparation, cleaning, and feature engineering steps were carried out employing pandas (version 1.2.3) and NumPy (version 1.19.2). The scikit-learn library (version 0.24.1) was the foundation for model development, encompassing the entire modeling pipeline from pre-processing to tuning and evaluation [29–31]. The model-building process is outlined in Fig 2 below.

## Results

We analyzed data of 41,394 PLHIV with 266,520 observations receiving treatment across four Nigerian states, with an IIT rate of 33.7% (Adamawa: 17.1%; Akwa Ibom: 38.7%; Cross River: 17.7%; Niger: 42.4%). Akwa Ibom state accounted for over half (52.9%) of the study population, followed by Niger (16.9%), Adamawa (15.5%), and Cross River (14.7%) states. About two-thirds of the study population were women (65.4%) and in a marital union (63.2%). More than half were between the ages of 25–39-year-old (52.5%), had senior secondary school education (54.2%) and were unemployed (58.4%) at the time of enrolment into care. Table 2 presents an overview of the essential socio-demographic characteristics of the study population.

**Table 2:** Basic socio-demographic characteristics of the study population.

Variables	N = 41,394 (%)
**Age (Years)**	
0 - 9	1,220 (2.91)
10 - 14	499 (1.21)
15 - 24	5,096 (12.31)
25 - 39	21,731 (52.50)
40 +	12,848 (31.04)
**Sex**	
Female	27,069 (65.39)
Male	14,325 (34.61)
**Marital Status**	
Single	12,878 (31.56)
Married	25,771 (63.15)
Divorced	407 (1.00)
Separated	648 (1.59)
Widowed	1,104 (2.71)
Missing	586 (1.4)
**Education**	
None	6,652 (16.75)
Primary	6,510 (16.39)
Junior Secondary	835 (2.10)
Senior Secondary	21,538 (54.23)
Post-Secondary	2,522 (6.35)
Quranic Education	1,659 (4.10)
Missing	1,678 (4.18)
**Employment Status**	
Unemployed	23,680 (58.42)
Employed	14,841 (36.61)
Student	1,914 (4.72)
Retired	101 (0.25)
Missing	858 (2.1)
**State**	
Adamawa	6,423 (15.52)
Akwa Ibom	21,892 (52.89)
Cross River	6,095 (14.72)
Niger	6,984 (16.87)

### Model results & evaluation

Our analysis found that the AdaBoost model performed better than the other two models across various performance metrics, such as sensitivity, F1-score, PR-AUC, and Matthews correlation coefficient (MCC) (Table 3). Specifically, the AdaBoost model had a higher sensitivity of 60.1%, indicating its ability to accurately identify positive cases, which is critical in predicting IIT. In addition, the AdaBoost model had the highest F1 score of 0.603, which suggests a well-balanced performance between precision and recall. This is important since the key objective is to minimize the impact of misclassifying positive cases, which, in this case, are IIT. Furthermore, the AdaBoost model achieved the highest PR-AUC value of 0.498, indicating superior performance across the threshold value. The AdaBoost model also had an MCC of 0.404, which was higher than the other models, indicating its superior performance in predicting IIT for an imbalanced outcome.

**Table 3:** Comparison of models performance metrics.

Models	Sensitivity^1^	Specificity^2^	PPV^3^	F1 score^4^	PR-AUC^5^	ROC-AUC^6^	MCC^7^
**Log Reg**	37.5%	89.0%	63.3%	0.471	0.447	0.743	0.314
**Random Forest**	39.0%	92.2%	71.7%	0.505	0.484	0.804	0.382
***AdaBoost***	60.1%	80.2%	60.5%	0.603	0.498	0.758	0.404

^1^Sensitivity: proportion of next missed drug refills (pharmacy visits) correctly identified.

^2^Specificity: proportion of next attended drug refills correctly identified.

^3^PPV (Positive Predictive Value): proportion of next drug refills predicted as missed that are truly missed.

^4^F1-score: harmonic mean of PPV and sensitivity.

^5^PR-AUC: measures PPV-Sensitivity trade-off.

^6^ROC-AUC: (Receiver Operating Characteristics Area Under the Curve) proportion of observations correctly classified.

^7^MCC: the ability to distinguish between the negative (non-IIT) and positive (IIT) when they are imbalanced.

The random forest model, however, had the highest specificity (92.2%), PPV (71.7%), and ROC-AUC (80.4%). Although the ROC-AUC metric indicates that the Random Forest model would be better overall (especially for the negative cases), the AdaBoost model was preferable over Random Forest due to its higher sensitivity and F1 score, which aligned better with accurately identifying IIT cases. Threshold-dependent metrics like sensitivity and PPV were also key considerations. The AdaBoost model better-balanced precision and recall for this imbalanced outcome, maintaining reasonable precision while significantly enhancing sensitivity.

Table 4 below presents the final AdaBoost model’s performance, following hyperparameter tuning, and its performance in predicting treatment interruptions by age and sex. The final AdaBoost model achieved the same sensitivity values for females and males, with 69.2%. However, the model exhibited a higher specificity for males at 83.7% compared to females at 81.7%. The positive predictive value was also higher for males at 67.2% compared to females at 66.0%. Overall, the model better predicted IIT for males, with a higher F1-score, PR-AUC, ROC-AUC, and MCC values than females.

**Table 4:** Comparison of AdaBoost model performance metrics by age and sex.

Features	Sensitivity^1^	Specificity^2^	PPV^3^	F1 score^4^	PR-AUC^5^	ROC-AUC^6^	MCC^7^
***Final***	69.2%	82.3%	66.4%	0.678	0.563	0.843	0.510
***By Sex***							
Female	69.2%	81.7%	66.0%	0.676	0.562	0.838	0.503
Male	69.2%	83.7%	67.2%	0.682	0.566	0.853	0.525
***By Age Group***							
0 - 9	52.9%	86.6%	64.7%	0.582	0.660	0.787	0.419
10 - 14	54.0%	82.8%	60.3%	0.569	0.658	0.783	0.379
15 - 24	70.1%	79.7%	65.1%	0.675	0.743	0.833	0.490
25 - 39	69.8%	81.6%	66.4%	0.681	0.743	0.840	0.508
40+	71.0%	83.9%	67.5%	0.692	0.758	0.863	0.542

^1^Sensitivity: proportion of next missed drug refills (pharmacy visits) correctly identified;

^2^Specificity: proportion of next attended drug refills correctly identified;

^3^PPV (Positive Predictive Value): proportion of next drug refills predicted as missed that are truly missed;

^4^F1-score: harmonic mean of PPV and sensitivity.

^5^PR-AUC: measures PPV-Sensitivity trade-off

^6^ROC-AUC: (Receiver Operating Characteristics Area Under the Curve) proportion of observations correctly classified

^7^MCC: the ability to distinguish between the negative (non-IIT) and positive (IIT) when they are imbalanced.

By age group, the model better predicted IIT for the age groups 15–24, 25–39, and 40 + , with sensitivity values ranging from 69.8% to 71.0%, specificity values ranging from 79.7% to 83.9%, and positive predictive values ranging from 65.1% to 67.5%. These age groups also observed the highest F1-score, PR-AUC, and MCC values. Notably, the model exhibited a lower sensitivity value for the age group 0–9, at 52.9%, indicating its lower ability to correctly identify positive cases in this age group. However, the model achieved a relatively high specificity value of 86.6% in this age group.

### Key predictors

**Fig 3:**
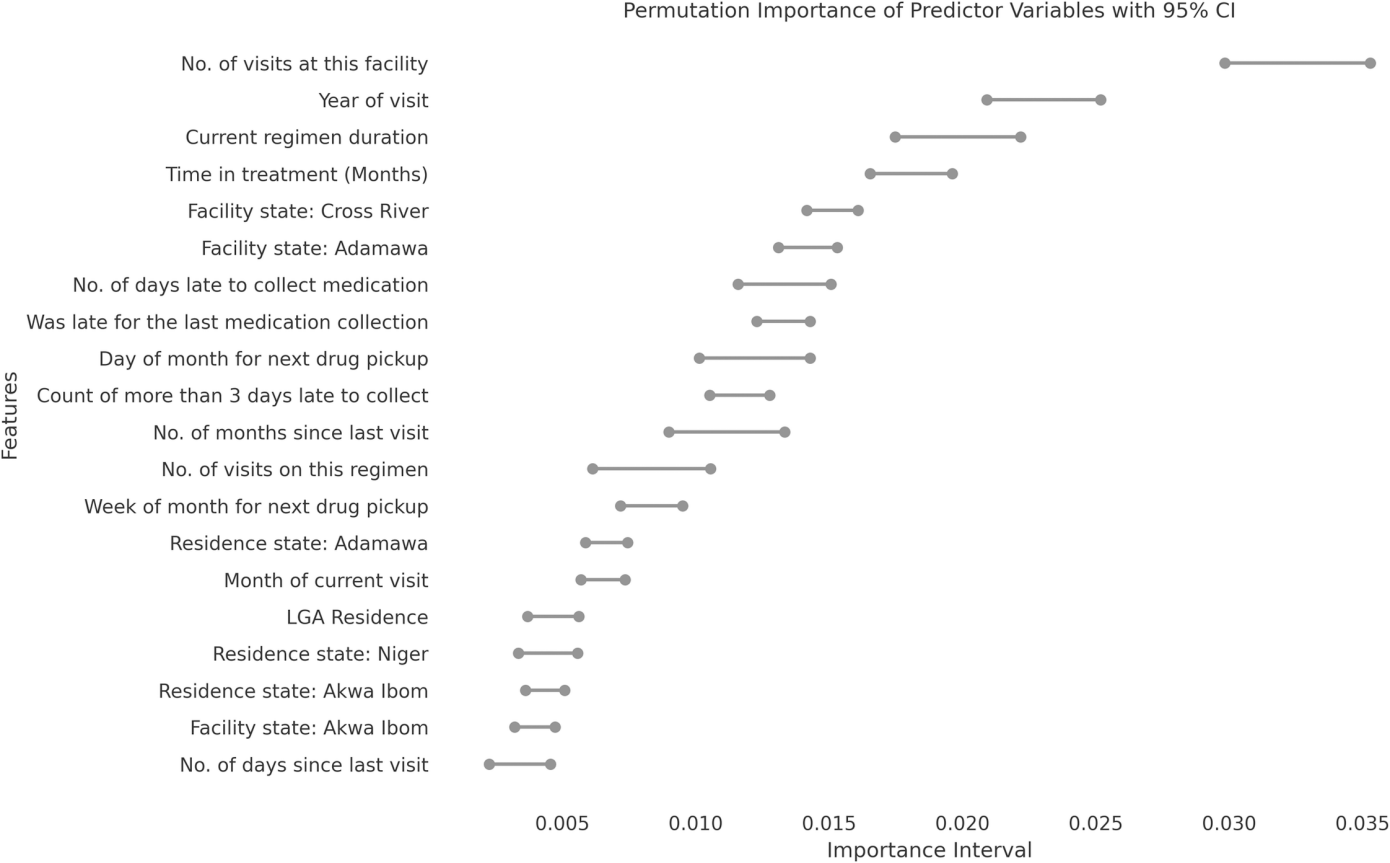
Permutation importance of top 20 most predictive variables.

Fig 3 illustrates the permutation importance of the critical predictors for the final AdaBoost Model. These are the top 20 features with the highest contribution to the model in predicting the risk of having an IIT. Our model relied on correlations in the PLHIV prior behavior and visit history (frequency of lateness, time in treatment, and time-based features), and geographical predictors, including the facility’s location and client’s place of residence, to segment the risk of the outcome. Viral load and demographic characteristics like age and sex had minimal predictive power.

## Discussion

This study explores the use of ML in predicting HIV continuity of care. Among the PLHIV included in the study, an IIT rate of 33.7% varied from 17.7% in Cross River state to 42.4% in Niger state. We found that the AdaBoost model performed best in predicting the risk of IIT among PLHIV and had the smallest probability of misclassifying positive cases with comparatively and cumulatively better performance metrics than the other two models. The final model could correctly predict IIT risk 69.2% percent of the time (sensitivity), specificity of 82.3%, PPV of 66.4%, and superior performance in predicting IIT for an imbalanced outcome (MCC = 0.51). The model better predicted IIT for males aged 15–24, 25–39, and 40 + . However, the model exhibited a lower sensitivity value for the age groups 0–9 and 10–14. PLHIV prior behavior and visit history, geographic predictors, and demographic features (to a much lesser degree) were key predictors of the risk of IIT.

Previous studies have examined the use of predictive analytics to identify PLHIV at high risk of IIT, and their findings support the performance metrics of our model. For example, Stockman et al., 2022., conducted a study in Nigeria using EMR data and reported a PR-AUC of 0.52 and MCC of 0.37, similar to our results of 0.56 and 0.51, respectively [32]. However, their results were obtained from an XGBoost model. Similarly, Ogbechie et al., 2023, used boosting classification algorithms to predict IIT among newly enrolled PLHIV in Nigerian facilities and reported higher performance metrics, including a sensitivity of 81%, specificity of 88%, and PPV of 83% [33]. In Tanzania, Fahey et al., 2022, employed decision trees to predict the risk of PLHIV disengagement within six months and reported a mean sensitivity of 54.7%, slightly lower than found in our analysis [34].

Additionally, Maskew et al., 2022, conducted a study across two districts in South Africa and reported a PR-AUC of 0.69 using an AdaBoost classifier model, which is close to our findings [17]. However, their PPV was much lower at 18%. Another study on Mozambican EMR data using a Random Forest model yielded PR-AUC of 0.65 and MCC of 0.45, aligning more closely with our results from the AdaBoost model [32].

Our model captured the non-linear interactions between features, and thus, the top 20 predictors should be considered as a group rather than individual causative factors. Clinical history, location, past behavior, next appointment date, and demographic characteristics were identified as top features in a similar machine-learning analysis in Nigeria, supporting our findings [32]. However, the order of importance differed in their study of Mozambican data, with past behavior, clinical history, demographics, next appointment date, and location being the most important features in descending order. Another study emphasized the importance of a PLHIV history of retention in care, appointments, and access to care in predicting the risk of IIT [35]. They also observed a decrease in the predictive importance of demographic factors as more robust data became available. The duration of treatment consistently emerged as a critical predictor of relative importance across these studies, including ours [32,35,36]. In contrast, Esra et al., 2023, found the demographic feature (age) to be the most crucial feature, with sex among the top predictive features in their data analysis from two South African provinces [37]. However, a similar analysis of data from two other South African provinces identified days late and visits as the most important features, with demographic factors (age and sex) playing a lesser role [37]. In another ML model, PLHIV history of clinic visits and attendance was the most significant predictor of missed visits, explaining nearly half of such occurrences [38]. This is particularly instructive, as almost half of our top 20 predictors are historical clinic attendance and visits variables.

Systematic reviews of studies using conventional statistical approaches have similarly highlighted demographic, behavioral, geographic, and clinical factors as important predictors of IIT risk [39–41]. Multiple studies have identified the duration of treatment as a significant predictor of both adherence and non-adherence to HIV treatment and the risk of IIT [42]. It has been argued that as PLHIV experience improved health over time, they may become complacent with adherence [43,44]. On the other hand, prolonged engagement with HIV care services, coupled with the associated health and well-being improvements, has been suggested to reinforce adherence by fostering trust in the healthcare system and commitment to maintaining positive health outcomes[45,46].

The history of clinic visits and behavior, from which most of the top predictor variables in our model were derived, including timeliness of clinic visits and missed appointments, has been associated with the risk of IIT and subsequent clinical outcomes [47,48]. It has been postulated that this is due to a wide variety of clinical, structural, and psychosocial issues, with Geng et al., 2011 particularly highlighting accessibility to care and logistics challenges in Sub-Saharan Africa [47,49,50]. Socioeconomic factors, such as education level, place of residence, and financial status, also play an important role in shaping ART visit patterns [41,51]. These socioeconomic factors often vary by context as individuals navigate competing daily demands [51]. For example, urban dwellers were at higher risk of IIT in a study conducted in the Democratic Republic of Congo [52]. However, a systematic review and meta-analysis of studies in Ethiopia by Gesesew et al., 2017 reported a higher risk of IIT among rural dwellers, emphasizing the importance of contextual considerations [53].

Geographic predictors from our top 20, like residence state, LGA residence, and facility state, may also influence the risk of IIT through contextual community-level socioeconomic determinants and facility-specific factors, including the availability and accessibility of care and clinic contact hours [41,42,54]. Additionally, facility-level determinants have been associated with the risk of IIT, including facility care levels, staffing, privacy concerns, inadequate documentation, and poor recordkeeping practices [41,42,55,56]. Notably, in Nigeria, health outcomes disparities, human resources for health imbalances, and socioeconomic inequalities have been identified as key drivers of health inequities across LGAs, states, and geopolitical zones [57,58].

Although findings from ML and conventional approaches overlap, ML’s focus on data-driven, accurate predictions contrasts with the relationship-centric inference of conventional methods [59,60]. Furthermore, complex computational techniques, such as Bayesian hierarchical frameworks, have shown superior performance compared to traditional frequentist methods in risk prediction [61].

Drawing on our findings, this model would benefit from integration into routine clinical workflows, continuous monitoring, and evaluation to validate its accuracy and assess its performance in real-world settings. Future research exploring how this model and similar ones perform across various healthcare systems, particularly in resource-limited settings, could offer valuable insights into the role of contextual factors, such as health system capacity, socioeconomic disparities, and policy environments, in shaping IIT risk.

### Strengths and limitations

We utilized a multi-center EMR system, which provides real-world evidence (RWE) for this study. This approach allowed for the evaluation of the natural history of the disease, treatment course, and standard of care across multiple settings, enhancing the generalizability of the findings. Additionally, the study included subgroup analyses based on gender and age groups, allowing for a more detailed understanding of these specific populations. However, our data has inherent limitations and biases. EMR data are typically incomplete and inconsistent [62]. There is often variability in the distribution of clients across the dataset influenced by factors such as access to care (e.g., economic class, sex, distance to the facility) and variations in service delivery.

Furthermore, there is significant bias in “hidden” data, where clients who discontinue treatment may require the most support but generate limited data or fewer longitudinally connected data points [62]. On the other hand, stable clients generate a lot of data, causing algorithms to be exact in identifying low-risk individuals. Additionally, ML may reflect human biases and exacerbate bias among disadvantaged populations [62]. Finally, this analysis is based on a retrospective observational design using routinely collected data. As such, we can only identify associations between various predictors and IIT and not determine causality.

## Conclusion

This study highlights the utility of ML in predicting the risk of IIT among PLHIV, with the AdaBoost model demonstrating superior performance. Key predictors—prior behavior, visit history and geographic factors should be interpreted collectively rather than as individual causative factors, and the traditional over-reliance on demographic attributes should be reduced. While ML models offer significant potential for improving predictive accuracy, further research is required to address data biases, disparities in healthcare access, and the contextual factors that shape IIT, particularly in resource-constrained settings.

## Supporting information

S1 Table List of engineered features. (DOCX)
