# Supplementary material for: Use of machine learning in predicting continuity of HIV treatment in selected Nigerian States
Source: PLOS Glob Public Health. 2025 Apr 24;5(4):e0004497. doi: 10.1371/journal.pgph.0004497 (PMC12021289; doi:10.1371/journal.pgph.0004497)
Supplement: S1 Table — (DOCX) [file pgph.0004497.s001.docx]

**List of Engineered Features**

| **Feature** | **Definition** | **Data Source** |
| --- | --- | --- |
| ***Age at Initiation*** | Age at start date of ART initiation | Patient |
| ***Age at Visit*** | Current age (years) | Patient/Pharmacy |
| ***Sex*** | Male or female | Patient |
| ***Days on ART (Time in treatment)*** | Number of days/month since initiation | Patient,Pharmacy |
| ***Visit count*** | Number of historical visits at facility | Pharmacy |
| ***Viral load count*** | Number of historical viral loads results recorded | Laboratory |
| ***ART regimen duration visits*** | Number of visits on current ART regimen | Laboratory |
| ***Current drug refill/visit: >3 days late*** | Was > 3 days late for current visit | Pharmacy,Clinic |
| ***Current drug refill/isit: >90 days late*** | Was > 90 days late for current visit | Pharmacy,Clinic |
| ***Current drug refill/visit: >28 days late*** | Was > 28 days late for current visit | Pharmacy,Clinic |
| ***Current drug refill/visit: start of month*** | Is current visit at the start of the month | Pharmacy,Clinic |
| ***Current drug refill/visit: week of month*** | Day of week of current visit (1-7) | Pharmacy,Clinic |
| ***Current drug refill/visit: >60 days late*** | Was > 60 days late for current visit | Pharmacy,Clinic |
| ***Current drug refill/visit: >7 days late*** | Was > 7 days late for current visit | Pharmacy,Clinic |
| ***Current drug refill/visit: end of month*** | Is current visit at the end of the month | Pharmacy,Clinic |
| ***Last drug refill/visit: day of month*** | Day of month of last visit (1-31) | Pharmacy,Clinic |
| ***Last drug refill/visit: day of week*** | Day of week of last visit (1-7) | Pharmacy,Clinic |
| ***Last drug refill/visit: month of year*** | Month of the year of last visit (1-12) | Pharmacy,Clinic |
| ***Last drug refill/visit: weekend*** | Was last visit on the weekend | Pharmacy,Clinic |
| ***Last VL Value*** | Results of last viral load recorded (copies/mL) | Laboratory |
| ***Late last drug refill/visit*** | Was patient late for last visit | Pharmacy,Clinic |
| ***Months since is drug refill/visit*** | Number of months since first visits | Pharmacy,Clinic |
| ***Months since last drug refill/visit*** | Number of days since last visits | Pharmacy,Clinic |
| ***Months since last drug refill/visit*** | Number of months since last attended visit | Pharmacy,Clinic |
| ***Next visit: start of month*** | Is next scheduled visits at the start of the month | Pharmacy,Clinic |
| ***Next visit: day of month*** | Day of month of next scheduled visit (1-31) | Pharmacy,Clinic |
| ***Next visit: day of week*** | Day of week of next scheduled visit (1-7) | Pharmacy,Clinic |
| ***Next visit: end of month*** | Is next scheduled visits at the end of the month | Pharmacy,Clinic |
| ***Next visit: quarter of year*** | Quarter of the year (starting from Jan 1) (1-4) | Pharmacy,Clinic |
| ***Next visit: week of month*** | Week of month of next scheduled visits (1-4) | Pharmacy,Clinic |
| ***Next visit: weekend*** | Is next scheduled visit on the weekend? | Pharmacy,Clinic |
| ***Next visit: Year*** | Year of next scheduled visit | Pharmacy,Clinic |
| ***Number of Days missed*** | Number of days for which no ART medication has been prescribed | Pharmacy,Clinic |
| ***Number of Months missed*** | Number of months for which no ART medication has been prescribed | Pharmacy,Clinic |
| ***Regimen line change between drug pickup*** | Indicator if a patient has changed regimen lines from previous visit (1 if True, False if not 0) | Pharmacy |
| ***Same LGA residence as facility*** | Indicator whether or not stated local governmental residence is the same as the facility LGA | Clinic |
| ***Stage Worsened*** | Indicator (1 if True,0 if False) weather or a patient has moved from Clinic Stage 1 to 2, 2 to 3,3 to 4 | Clinic |
| ***Status History Changed*** | Indicator (1 if True,0 if False) whether or not status has changed from ART START to ART_RESTART; LOSS TO_FOLLOW_UP; STOPPED TREATMENT, KNOWN DEATH | Patient |
| ***Virally Suppressed*** | Indicator for Viral Suppression whereby (1 if Suppressed <1000cp/mL, 0 if Unsuppressed) | Laboratory |
| ***Viral Suppression Categorization (including undetected)*** | Indicator *(0 if Undetectable [<= 400 cp/mL], 1 if Suppressed not Undetectable [>400CP/mL and <1000 cp/mL], 2 if Unsuppressed [> 1000cp/mL])* for Viral Load suppression. | Laboratory |
| ***Viral Load Type*** | Viral Load monitoring strategy (routine, targeted or unspecified) | Laboratory |
| ***Weight Loss*** | Whether or not there was a weight loss greater than 10% from previous clinic visit (1 if True,0 if False) | Clinic |
| ***28 days late count up until current visit*** | Number of visits attended >28 days late | Pharmacy |
| ***3 days late ratio*** | Ratio of historical visits attended > 3 days late vs. visits attended on time | Pharmacy |
| ***60 days late count*** | Number of visits attended >60 days late | Pharmacy |
| ***60 days late ratio*** | Ratio of historical visits attended > 60 days late vs. visits attended on time | Pharmacy |
| ***7 days late count*** | Number of visits attended >7 days late | Pharmacy |
| ***7 days late ratio*** | Ratio of historical visits attended > 3 days late vs. visits attended on time | Pharmacy |
